# Supplementary material for: Blood pressure variability and night-time dipping assessed by 24-hour ambulatory monitoring: Cross-sectional association with cardiac structure in adolescents
Source: PLoS One. 2021 Jun 16;16(6):e0253196. doi: 10.1371/journal.pone.0253196 (PMC8208567; doi:10.1371/journal.pone.0253196)
Supplement: Attachment — Submitted filename: Response_to_reviewers.docx [file pone.0253196.s002.docx]

## We thank the reviewers for their comments on our manuscript titled “Blood pressure variability and night-time dipping assessed by 24-hour ambulatory monitoring: cross-sectional association with cardiac structure in adolescents”. We provide a point-by-point response below, with line numbers referred to from the tracked changes version of the manuscript attached.

**Reviewer #1: Goudswaard et al. explored the associations between several blood pressure measurements (clinic, 24-hours, 24-hour variability, night dipping) with echocardiographic indexes of cardiac structure in a population of adolescents. They found that systolic blood pressure, diastolic blood pressure and blood pressure variability are associated with RWT. Systolic blood pressure is also associated with systolic with LVMi2.7. The importance of exploring these associations in a population of adolescent is relevant, being primary prevention a fundamental tool nowadays to prevent healthcare system burdening. However, the paper has some issues that should better be addressed.**

We appreciate the encouraging comments regarding the relevance of the study. We will address the specific issues raised by the reviewers below.

**Major**

**As stated in the limitations, the rather small sample size might limit the relevance of the findings. Indeed, the healthy phenotype represented in the cohort might further hinder the appreciation of a concrete effect. The cross-sectional setting is another major limitation, as recognized by the authors. The associations are not strong and, from a clinical point of view, their relevance is mild.**

**Moreover, the discussion is rather plain and sometimes unspecific (e.g. line 352 “some associations”, lines 367-368 “adverse cardiovascular health” is a quite strong claim but it is not substantiate enough in the text, lines 380-385 are vague and do not draw a conclusion, maybe they should be partially shifted to methods) not emphasizing enough the main findings of the study and not providing neither a definite pathophysiologic hypothesis nor conveying a clear clinical message.**

We appreciate there are limitations such as a small sample size. This limitation has been addressed by acknowledging a potential lack of power to detect associations. However, as it is a cohort, we cannot increase our sample size. Moreover, this is currently the largest available data source with such detailed cardiovascular assessment in an adolescent population. To address this, we have expanded on the power limitation within the manuscript on line 459:

“The current study has several limitations. It is possible that our study may have lacked statistical power to detect some associations between BP variability and dipping and cardiac structure independently of mean BP due to a modest sample size. Despite this limitation, this study provides highly detailed measurements from each participant, and is still comparatively a large cohort size (compared to other studies which utilize ABPM and echocardiography techniques).”

The inclusion of healthy adolescents provides a novel aspect to the study, as many studies have only focused on patients with hypertension, who are extremely unusual in this age group. Indeed, the findings that average BP and BP variability are associated with cardiac remodeling even in those without hypertension is important as this would not currently be considered in those with a normotensive BP. We have put the findings into context in the discussion from line 401:

“Previous studies indicate that risk of subclinical organ damage is raised in participants who are pre-hypertensive, reinforcing the importance to consider BP as a risk factor even in those within the “physiologic” BP range. Further, these findings support the notion that the influence of BP on cardiac structure may begin early in life and that earlier risk assessment in adolescence may help avoid subclinical organ damage.”

We agree that the cross-sectional setting is a limitation, and we believe we have already addressed this appropriately within the limitations section.

The magnitude of the estimates may be still clinically relevant even if they are not particularly large. For example, a 1 SD increase in diastolic ARV was associated with a 0.13 SD increase in RWT. It is therefore possible that this change in RWT could recategorize a participant from having normal left heart geometry to having geometric remodeling. Furthermore, without intervention during adolescence and early adulthood, these differences might be expected to further widen at older ages, emphasizing the need for prevention. We have now addressed the effect size in the discussion on line 417:

“The magnitude of increase in RWT as a result of higher DBP variability could shift the participant’s category from being considered to have normal left heart geometry to having geometric remodeling. Furthermore, without intervention during adolescence and early adulthood, these differences might be expected to further widen at older ages, emphasizing the need for prevention.”

As suggested by both reviewer 1 and reviewer 2, we have replaced the use of “adverse cardiovascular health” to cardiac remodeling risk. We have also now avoided the use of “some associations” in order to be more specific. The sentences which were on lines 380-385, that compared methods of BP variability, have been removed as we agree this is not essential for the current study.

We have rewritten the discussion to make it more focused, ensuring that we have included suggestions of pathophysiologic mechanisms and clarifying the clinical message. This is now included on line 417:

“The magnitude of increase in RWT as a result of higher DBP variability could shift the participant’s category from being considered to have normal left heart geometry to having geometric remodeling. Furthermore, without intervention during adolescence and early adulthood, these differences might be expected to further widen at older ages, emphasizing the need for prevention. It has been suggested that higher BP variability may lead to organ damage by reduced ability of baroreceptors to modulate blood pressure or due to increased arterial stiffness.”

**The topic, i.e. primary/primordial prevention, even more in youth, is relevant so the findings deserve to be discussed, but with a more convincing tone. I believe that the authors should meaningful reassess the discussion: first briefly summarizing their main results and then discussing them stressing the importance of earlier prevention/correct risk assessment in adolescence to avoid subclinical organ damage in older age and the importance of not underestimate values within the “physiologic” range (see, for example, PMID: 32936272, PMID: 29908829), maybe contextualizing them with what observed in the current literature (e.g. with some numeric direct confrontation from what observed in other studies in adults).**

We appreciate the comment and have edited the discussion to incorporate suggestions. We have added to the discussion after summarizing associations between measures of average BP and cardiac structures. We have emphasized the need to consider those who may be pre-hypertensive but within the physiologic BP range when considering subclinical organ damage (with a reference to the study PMID 29908829 that shows this in adults), as well as indicated that these associations between average BP and cardiac structure are beginning early in life and therefore risk assessment from adolescents could be important.

From line 401, the discussion reads:

“Previous studies indicate that risk of subclinical organ damage is raised in participants who are pre-hypertensive, reinforcing the importance to consider BP as a risk factor even in those within the “physiologic” BP range. Further, these findings support the notion that the influence of BP on cardiac structure may begin early in life and that earlier risk assessment in adolescence may help avoid subclinical organ damage.”

**Minor**

**•Title: the title is quite generic and not informative of the results of the study.**

We have not altered the title of the manuscript as we are aware that some journals do not like the results to be presented in the title. If the editors felt it would be more appropriate, we would be happy to amend the title to “Cross-sectional evidence from adolescents that blood pressure and blood pressure variability are associated with cardiac structure.”.

**•Methods: as stated above, the sample size is rather small. I would appreciate a sample size analysis included in the paper.**

As this is a cohort study utilizing pre-existing data, the sample size is out of our control. We believe that examining and presenting the confidence intervals around our estimates is more correct and meaningful than post-hoc power calculations, which can be misleading (<http://dx.doi.org/10.1136/gpsych-2019-100069>), will always reflect the information presented in a confidence interval, and emphasise an unhelpful dichotomisation of ‘significant versus non-significant’ (https://www.bmj.com/content/322/7280/226.1).

**•Methods: ethnicity has not been adopted as confounder, though it is related with both blood pressure variability and cardiac structure (e.g. PMC7670766, PMID: 19578033, PMID: 31072636).**

We appreciate that ethnicity is an important confounder. Within the study we report that participants are of European ancestry, therefore ethnicity is not a confounding factor. This is mentioned within the limitations on line 466, but we have also added it to the results section on line 261.

**•Results. Mean population BMI of the population should be reported.**

The mean population BMI is important to report as it provides useful information about the study population. We have now added the mean population BMI to the results section to line 270, this was already reported in S1b Table.

**•Figure 2. Though most the associations are found with RWT, Figure 2 only shows LVMi. RWT should be implemented here.**

This is a helpful comment, we have updated Figure 2 so that it includes both LVMi (Fig 2A) and RWT (Fig 2B).

**Reviewer #2: The Authors explored the relationship between blood pressure (BP) variability and dipping and left ventricular (LV) remodeling in 587 adolescents (mean age 18 years). The main findings are that an increased BP variability is associated with higher relative wall thickness, and then concentric LV remodeling, even after adjustment for mean BP. The Authors conclude that "Measurement of BP variability might benefit cardiovascular risk assessment in adolescents". These results are quite novel and the results are reasonable. On the other hand, there are some points to consider.**

We thank the reviewer for their comments that the results are novel and reasonable. We will address the specific points below.

**The possible mechanisms relating increased BP variability to LV remodeling should be evaluated in detail.**

Apologies for not addressing this, we understand the importance of addressing potential mechanisms based on study findings. We have now included suggestions of mechanisms that may be leading from increased BP variability to LV remodeling. From line 422:

“It has been suggested that higher BP variability may lead to organ damage by reduced ability of baroreceptors to modulate blood pressure or due to increased arterial stiffness. However, as we did not assess causality, we cannot rule out that cardiac remodeling may be causing the increase in blood pressure variability. Further studies are warranted to explore mechanisms and direction of causality.”

**The relatively small cohort size (particularly considering the usually initial LV remodeling in adolescents) and the lack of longitudinal data are intrinsic limitations of this study, which should be acknowledged and discussed.**

We have now acknowledged the relatively small cohort size within the limitations. We initially mentioned a potential lack of power to detect associations, and have expanded on this. We now mention that this is due to a modest sample size, although in comparison to other studies that include similar ABPM and echocardiographic information, that the sample size is actually quite large. From line 459 in the limitations, it now reads:

“The current study has several limitations. It is possible that our study may have lacked statistical power to detect some associations between BP variability and dipping and cardiac structure independently of mean BP due to a modest sample size. Despite this limitation, this study provides highly detailed measurements from each participant, and is still comparatively a large cohort size (compared to other studies which utilize ABPM and echocardiography techniques). The lack of longitudinal data is also a limitation; following up the BP variability and cardiac structure at older ages will enable more detailed analyses of how these processes develop over time”

**To adjust for mean BP, you should exclude multicollinearity between metrics of BP variability and mean BP. I cannot find this evaluation in your Methods section.**

We have evaluated the potential for collinearity between mean BP and BP variability. Correlations between mean BP and BP variability were low to moderate, ranging from 0.01 to 0.36, and we are therefore confident that collinearity is not a concern in these analyses. We have detailed these correlations in the methods and results sections of the manuscript from line 216: “To verify analyses were not affected by collinearity, we assessed correlations between measures of mean BP and measures of BP variability.” and in the results from line 374: “Correlations between mean BP and BP variability were low to moderate, with the correlation coefficient (r) ranging from 0.01 to 0.36 (S6 Table), suggesting collinearity is not a concern.”. We have also provided the correlations as S6 Table.

**I would not speak of cardiovascular risk assessment, but rather of the evaluation of the risk for cardiac remodeling.**

This is a helpful comment and we agree that although cardiac structures have been shown to relate to cardiovascular risk, it is better to comment on cardiac remodeling. Where relevant, we have replaced “cardiovascular risk” with “cardiac remodeling risk”.
